# *ESR1* Y537S Detected in a Brain Metastasis but Not in Plasma ctDNA in HR+/HER2-Low Metastatic Breast Cancer: A Case Report

**DOI:** 10.3390/curroncol33080454

**Published:** 2026-07-28

**Authors:** Aemélia Nègre, Lorène Seguin, Arthur Géraud Cremieux, Frederic Viret, Agnès Tallet, Cornel Popovici, Anthony Gonçalves, Alexandre de Nonneville

**Affiliations:** 1 Department of Medical Oncology, Institute Paoli Calmettes, 13009 Marseille, France; 2 Faculty of Medicine, Aix Marseille University, La Timone Campus, 13005 Marseille, France; 3 Department of Radiotherapy, Institute Paoli Calmettes, CRCM, 13009 Marseille, France; 4 Cancer Biology Department, Institute Paoli Calmettes, 13009 Marseille, France

**Keywords:** breast cancer brain metastases, *ESR1* mutation, circulating tumor DNA, CNS progression, case report

## Abstract

Brain metastases in hormone receptor-positive metastatic breast cancer remain incompletely characterized at the molecular level. We report isolated central nervous system progression in a 60-year-old woman with hormone receptor-positive breast cancer initially diagnosed at the age of 38 and metastatic recurrence diagnosed at the age of 55. After approximately five years of extracranial disease control with letrozole plus palbociclib, she developed multiple brain metastases, including a large cerebellar lesion requiring surgical resection. Molecular profiling of the resected lesion identified *ESR1* Y537S and *ERBB2* Y772_A775dup, whereas postoperative plasma circulating tumor DNA analyzed using a sensitive next-generation sequencing assay showed no detectable somatic alteration. Extracranial disease remained in complete metabolic response. This tissue–plasma discordance is compatible with spatial genomic heterogeneity but may also reflect a low circulating tumor fraction, postoperative reduction in tumor burden, and limited release of tumor-derived DNA from CNS lesions into the systemic circulation. This case highlights the limitations of plasma circulating tumor DNA for evaluating CNS-limited progression and supports direct molecular profiling of brain metastases when tissue is available.

## Simple Summary

Breast cancer can progress in the brain even when disease elsewhere remains well controlled. We report a woman whose hormone-sensitive breast cancer remained controlled outside the brain during long-term treatment but who developed several brain metastases. Genetic analysis of a surgically removed cerebellar metastasis identified a mutation in the estrogen receptor gene associated with resistance to aromatase inhibitors. The same mutation was not detected in a blood sample using a highly sensitive test. This discrepancy may reflect limited release of tumor DNA from brain lesions into the bloodstream and does not prove that the mutation was present only in the brain. This case shows that a negative blood-based tumor DNA result cannot exclude isolated brain progression and that direct analysis of brain metastasis tissue can provide clinically useful information when surgical material is available.

## 1. Introduction

Brain metastases represent a major clinical challenge in metastatic breast cancer (MBC), particularly because of their distinct biology and limited sensitivity to systemic therapies due to the differences in drug bioavailability imposed by the blood–brain barrier. Approximately 15–25% of patients with breast cancer will develop brain metastases, with marked differences according to molecular subtype [1].

While HER2-positive and triple-negative tumors have the highest predilection for central nervous system (CNS) spread, hormone receptor-positive (HR+) disease accounts for a non-negligible proportion of cases, mostly late in the natural history of the disease [2].

Metastatic dissemination is known to be accompanied by genomic diversification, with late metastatic sites often diverging from both the primary tumor and other metastatic deposits. This evolutionary phenomenon reflects acquisition of resistance mutations under treatment pressure, and contributes to clinical heterogeneity of therapeutic responses.

Among key resistance mechanisms in HR+ MBC, *ESR1* ligand-binding domain mutations (notably Y537S, Y537N, and D538G) are well-established drivers of endocrine resistance. Their prevalence increases with cumulative exposure to aromatase inhibitors. While common in visceral and bone metastases, *ESR1* mutations remain exceptionally rare in brain metastases, with only a few documented cases [3].

Importantly, molecular profiling of brain metastases often reveals alterations not detectable in circulating tumor DNA (ctDNA), reflecting both anatomical compartmentalization and biological isolation of CNS lesions [4]. Therefore, matched tissue and liquid biopsy analyses can provide complementary insights.

A previously published case report described a 37-year-old woman with hormone receptor-positive, HER2-negative metastatic breast cancer and isolated leptomeningeal progression during aromatase inhibitor (AI) treatment, in whom an *ESR1* activating mutation was detected in free tumor DNA derived from cerebrospinal fluid (CSF), but not in the circulating tumor DNA (ctDNA) derived from matched plasma samples [5].

Here, we report a case of isolated CNS progression in a patient with HR+/HER2-low metastatic breast cancer treated with an aromatase inhibitor plus a CDK4/6 inhibitor. An *ESR1* Y537S mutation was detected in the resected cerebellar metastasis but was not identified in postoperative plasma ctDNA analyzed using the Hedera DX HP2 assay.

This case illustrates tissue–plasma molecular discordance during isolated intracranial progression and highlights the complementary diagnostic value of tissue-based sequencing when plasma ctDNA is negative.

## 2. Method

This case report was conducted in accordance with the CARE (CAse REport) guidelines to ensure transparent and complete reporting of clinical information. Written informed consent for publication, including the use of clinical data and imaging, was obtained from the patient prior to submission. The patient was informed of the purpose of the report and agreed to the anonymous use of her medical information for scientific and educational purposes. A completed CARE checklist has been provided as Supplementary Information. GenAI v5.5 was used only for limited language editing to improve grammar, syntax, and English fluency. It was not used to generate or modify any scientific content, data, analyses, figures, or interpretations.

## 3. Case Report

A 38-year-old woman was diagnosed in 2003 with a grade II NST invasive carcinoma of the right breast (cT2N0), which was HR-positive and HER2-negative. She underwent surgery followed by adjuvant radiotherapy and tamoxifen, which was discontinued after 1.5 years due to intolerance.

In 2020, 17 years after the initial diagnosis, at the age of 55, she developed a locoregional and pulmonary metastatic recurrence (ER 100%, PR 10%, HER2 IHC 2+/DISH-negative). Letrozole plus palbociclib was initiated, resulting in a complete metabolic response. In June 2021, while extracranial disease remained controlled, she underwent mastectomy with immediate breast reconstruction. The complete extracranial metabolic response was subsequently maintained for approximately four years.

Molecular profiling of tumor tissue was performed using an in-house targeted hybrid-capture 96-gene NGS panel (ISO15189-accredited) [6], enabling detection of single-nucleotide variants, insertions/deletions, and copy number alterations. This analysis identified an *ERBB2* Y772_A775dup in-frame duplication with a variant allele frequency (VAF) of 15%.

In October 2022, the patient underwent serial ctDNA monitoring every 3 months using Guardant360 CDx (Guardant Health, Inc., Palo Alto, CA, USA), with no detectable mutations until July 2024.

**Figure 1:**
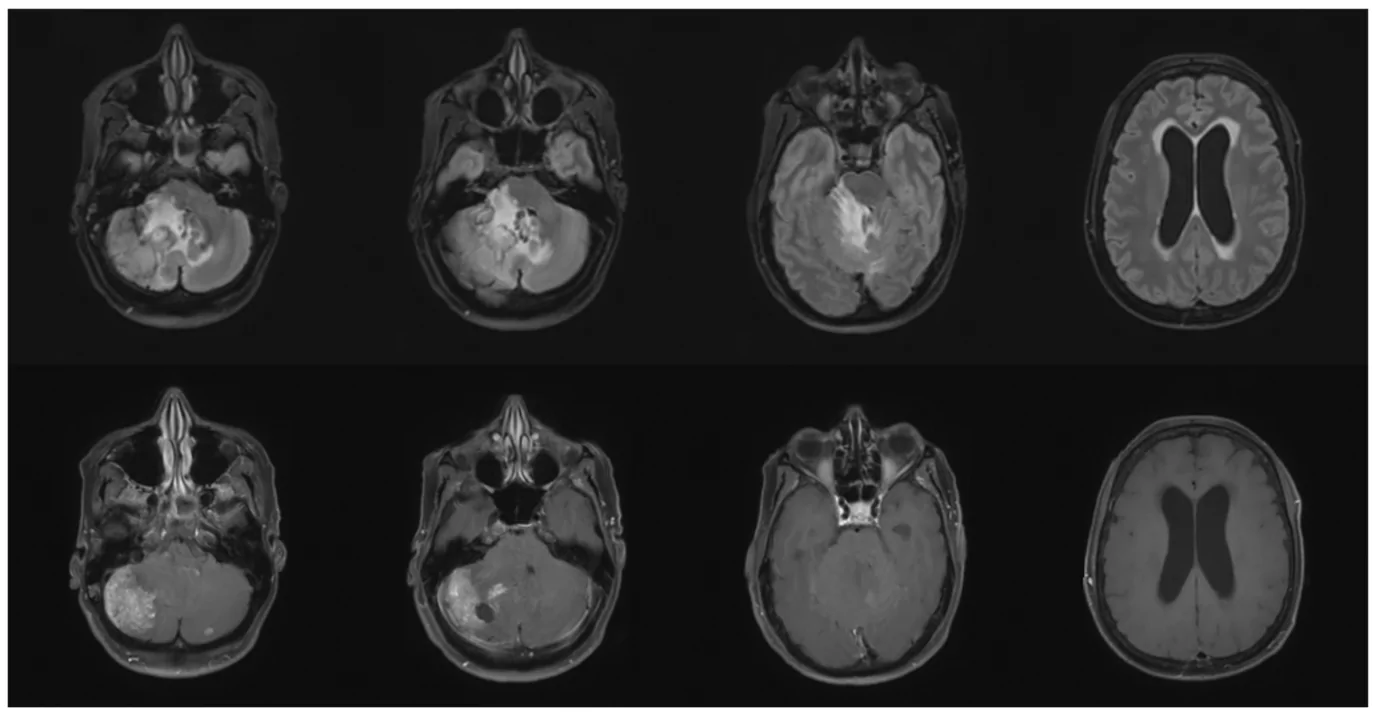
Brain MRI at diagnosis of neurological symptoms: **Upper row**: axial T2-FLAIR sequences. **Lower row**: axial T1-weighted sequences after gadolinium injection.

Beginning in September 2024, she developed progressive vertigo and blurred vision, which worsened by April 2025. Neurological examination in May 2025 revealed right-sided dysmetria and a broad-based gait. A brain MRI revealed several infracentimetric supratentorial lesions, along with a large right cerebellar lesion with mass effect and obstructive hydrocephalus (Figure 1). A ventriculoperitoneal shunt was placed emergently, followed by surgical resection of the cerebellar mass. A whole-body PET scan confirmed persistent complete extracranial response, with no visceral or bone activity.

The resected lesion was confirmed as metastatic breast adenocarcinoma, with ER expression of 100%, PR expression of 80%, and a HER2 IHC score of 1+. Although the HR-positive/HER2-negative phenotype was preserved, PR expression was higher than in the 2020 recurrence (80% versus 10%).

A subsequent molecular analysis of the resected tumor tissue, performed on the in-house targeted NGS panel, revealed the following alterations:

- An already-identified variant in the *ERBB2* gene (Y772_A775dup), with a VAF of 59%.
- A pathogenic variant in the *ESR1* gene (Y537S) located in exon 8, with a VAF of 40%, known to be a canonical activating mutation driving complete ligand-independent ER activation and strong AI resistance.
- A low-level amplification (8 to 10 copies) of the genomic region encompassing the *CCND1* locus.

To evaluate whether the *ESR1* alteration was detectable in the systemic circulation, postoperative plasma collected on 8 August 2025 was analyzed using the Hedera profiling 2 (HP2) ctDNA assay (Hedera Dx, Epalinges, Switzerland), a hybrid-capture 32-gene NGS panel designed to detect single-nucleotide variants, insertions/deletions, copy number alterations, and selected structural variants [7]. The assay covers clinically relevant *ESR1* ligand-binding domain hotspots, including Y537S/N/C, D538G, and E380Q, and has an approximate limit of detection of 0.1–0.5% variant allele frequency for established hotspot mutations.

No somatic alteration was detected in postoperative plasma, including *ESR1* Y537S or the previously identified *ERBB2* Y772_A775dup in-frame duplication. No copy number alteration was identified. This negative result was compatible with a very low circulating tumor fraction but did not establish that the alterations were confined to the CNS.

Following confirmation of isolated cerebral progression, multidisciplinary treatment included stereotactic radiotherapy to the resection cavity and the remaining intracranial lesions. Endocrine therapy was switched to fulvestrant, based on its expected higher activity against *ESR1*-mutated hormone-receptor-positive disease compared to letrozole [8]. The presence of an *ESR1* Y537S mutation further supported discontinuation of aromatase inhibitor therapy given the strong ligand-independent ER activation associated with this mutation. Palbociclib was replaced by abemaciclib, the latter being selected both for its potential CNS penetration [9] and its documented activity in combination with fulvestrant in the context of disease progression on AI plus CDK4/6 inhibitors [10].

**Figure 2:**
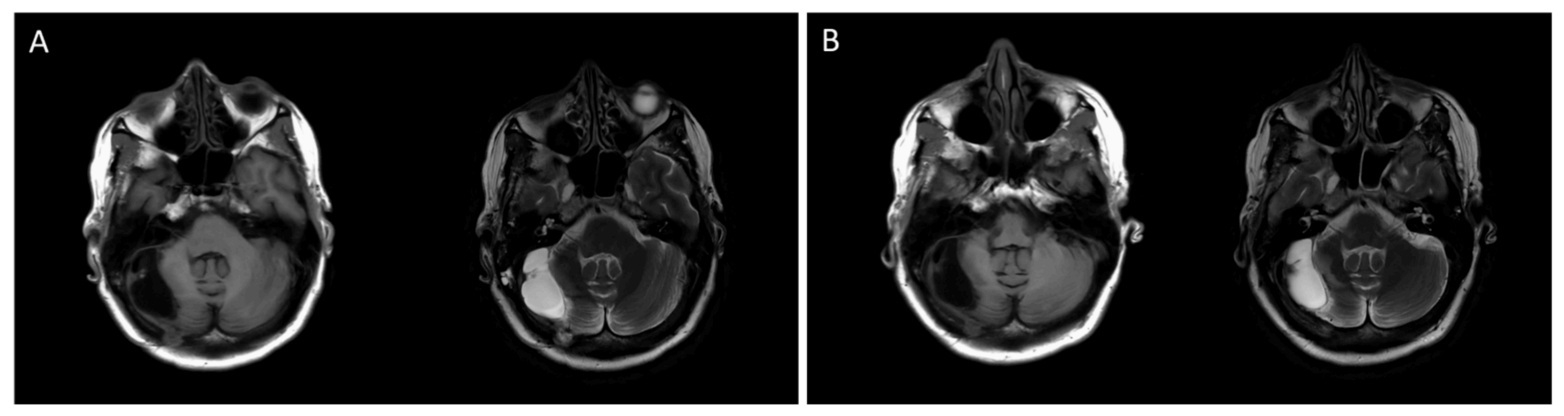
Follow-up brain MRI after local and systemic treatment. (**A**) November 2025 and (**B**) February 2026. At each time point, contrast-enhanced axial T1-weighted imaging is shown on the left and axial T2-weighted imaging on the right. Post-treatment findings remained stable without radiographic intracranial progression.

Imaging reassessments performed in November 2025 and February 2026, approximately 3 and 6 months after treatment modification, demonstrated a persistent complete extracranial metabolic response on PET imaging and stable intracranial findings on brain MRI (Figure 2).

## 4. Discussion

**Figure 3:**
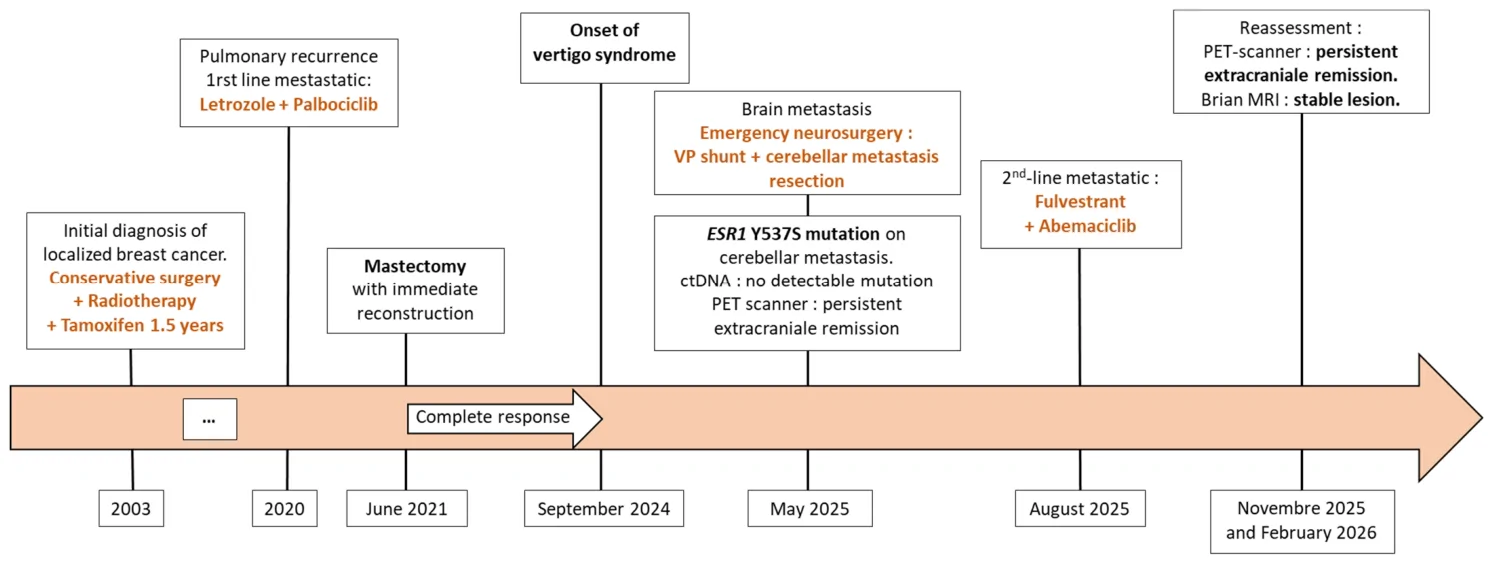
Clinical timeline summarizing the patient’s disease course, treatments, and molecular findings. The diagram outlines key events from the initial diagnosis of localized breast cancer in 2003 to the metastatic recurrence in 2020, followed by a durable complete response under letrozole plus palbociclib. The patient subsequently developed isolated neurological symptoms in late 2024, leading to the discovery of cerebellar and supratentorial brain metastases in May 2025. Surgical resection revealed an *ESR1* Y537S mutation in the analyzed brain metastasis, whereas postoperative plasma ctDNA showed no detectable somatic alteration and whole-body PET confirmed persistent extracranial metabolic response. Fulvestrant plus abemaciclib was initiated after neurosurgical and radiotherapeutic management, with stable intracranial findings and persistent extracranial metabolic response at 3- and 6-month follow-up.

This case illustrates several important biological principles (a summary of the patient’s clinical course and treatments is provided in Figure 3).

First, the detection of *ESR1* Y537S in the brain metastasis after prolonged exposure to an aromatase inhibitor is compatible with treatment-driven clonal selection and spatial heterogeneity. However, the available tissue and plasma analyses do not establish that the mutation was confined to the CNS [11].

Second, brain metastases may undergo independent evolutionary trajectories, resulting in molecular divergence from extracranial disease [12,13]. Accordingly, tissue sequencing remains essential when isolated CNS relapse is identified and surgical material is available.

Third, plasma ctDNA may remain negative in patients with CNS-limited progression, emphasizing that a negative ctDNA result does not exclude clinically relevant intracranial disease activity [14,15]. This is of particular importance in the context of emerging ctDNA-guided strategies aiming to adapt treatment intensity and imaging schedules in metastatic breast cancer. In HER2-positive disease, MRD-driven approaches using personalized ctDNA assays are being evaluated to support treatment de-escalation, while in HR-positive disease, trials such as PADA-2 investigate ctDNA dynamics to guide therapeutic modifications. However, this case highlights a key limitation of such approaches, as ctDNA may remain undetectable in the setting of isolated CNS progression. Therefore, reliance on ctDNA alone may be insufficient, and dedicated brain imaging should be maintained when clinically indicated.

The absence of *ESR1* Y537S detection in plasma ctDNA does not constitute definitive evidence that the mutation was confined to the CNS. Plasma sampling was performed after resection of the dominant cerebellar lesion, which may have further reduced tumor-derived DNA shedding. The observed discordance may therefore reflect a combination of a low circulating tumor fraction, postoperative reduction in intracranial tumor burden, and limited release of tumor-derived DNA from CNS lesions into the systemic circulation because of biological compartmentalization and the blood–brain barrier.

From a therapeutic perspective, oral selective estrogen receptor degraders represent a potential future options in *ESR1*-mutated disease, although their role in isolated CNS progression remains undefined [16,17,18,19]. Imlunestrant has shown promising activity and brain penetration in preclinical models [20,21,22], but clinical evidence in this specific setting remains to be established. The relative enrichment of the *ERBB2* Y772_A775dup variant in the brain metastasis is hypothesis-generating but does not establish a causal role in CNS progression. Trastuzumab deruxtecan could represent a subsequent treatment option based on the HER2-low phenotype and its reported intracranial activity; however, the predictive significance of this *ERBB2* alteration in the present setting remains uncertain.

Beyond endocrine resistance, preclinical data suggest that *ESR1* Y537S may influence the tumor microenvironment through YAP1-mediated fibroblast activation and conversion into cancer-associated fibroblasts [23]. Although this mechanism cannot be inferred from the present clinical observation, these findings provide a potential biological framework for future investigation.

## 5. Conclusions

This case describes isolated CNS progression in a patient with HR+/HER2-low metastatic breast cancer and sustained extracranial disease control. *ESR1* Y537S was detected in the resected cerebellar metastasis but not in postoperative plasma ctDNA. Although this discordance is compatible with spatial genomic heterogeneity, plasma negativity cannot establish that the mutation was confined to the CNS and may also reflect low circulating tumor fraction, postoperative reduction in tumor burden, and limited ctDNA shedding from intracranial lesions. This observation highlights the limitations of plasma liquid biopsy in CNS-limited clinical progression and supports an integrated diagnostic approach combining imaging and direct molecular profiling of metastatic tissue when available.

## Data Availability

All relevant data supporting the findings of this case report are included within article. Additional clinical data, including original MRI images, are not publicly available due to patient privacy and ethical restrictions.

## Supplementary Materials

The following supporting information can be downloaded at: https://www.mdpi.com/article/10.3390/curroncol33080454/s1, CARE Checklist of information to include when writing a case report.
