# Supplementary material for: ESR1 Y537S Detected in a Brain Metastasis but Not in Plasma ctDNA in HR+/HER2-Low Metastatic Breast Cancer: A Case Report
Source: Curr Oncol. 2026 Jul 28;33(8):454. doi: 10.3390/curroncol33080454 (PMC13510483; doi:10.3390/curroncol33080454)
Supplement: Supplementary file 1 [file curroncol-33-00454-s001.zip › curroncol-4395985-supplementary.pdf]

CARE Checklist of information to include when writing a case report

Topic	Item	Checklist item description	Reported on Line
**Title**	1	The diagnosis or intervention of primary focus followed by the words "case report"	✓
**Key Words**	2	2 to 5 key words that identify diagnoses or interventions in this case report, including "case report"	✓
	3a	Introduction: What is unique about this case and what does it add to the scientific literature?	✓
**Abstract (no references)**	3b	Main symptoms and/or important clinical findings	✓
	3c	The main diagnoses, therapeutic interventions, and outcomes	✓
	3d	Conclusion—What is the main "take-away" lesson(s) from this case?	✓
**Introduction**	4	One or two paragraphs summarizing why this case is unique (**may include references**)	✓
**Patient Information**	5a	De-identified patient specific information	✓
	5b	Primary concerns and symptoms of the patient	✓
	5c	Medical, family, and psycho-social history including relevant genetic information	✓
	5d	Relevant past interventions with outcomes	✓
	6	Describe significant physical examination (PE) and important clinical findings	✓
**Timeline**	7	Historical and current information from this episode of care organized as a timeline	✓
**Diagnostic Assessment**	8a	Diagnostic testing (such as PE, laboratory testing, imaging, surveys)	✓
	8b	Diagnostic challenges (such as access to testing, financial, or cultural)	✓
	8c	Diagnosis (including other diagnoses considered)	✓
	8d	Prognosis (such as staging in oncology) where applicable	✓
	9a	Types of therapeutic intervention (such as pharmacologic, surgical, preventive, self-care)	✓
	9b	Administration of therapeutic intervention (such as dosage, strength, duration)	✓
	9c	Changes in therapeutic intervention (with rationale)	✓
	10a	Clinician and patient-assessed outcomes (if available)	✓
	10b	Important follow-up diagnostic and other test results	✓
	10c	Intervention adherence and tolerability (How was this assessed?)	✓
	10d	Adverse and unanticipated events	✓
	11a	A scientific discussion of the strengths AND limitations associated with this case report	✓
	11b	Discussion of the relevant medical literature **with references**	✓
	11c	The scientific rationale for any conclusions (including assessment of possible causes)	✓
	11d	The primary "take-away" lessons of this case report (without references) in a one paragraph conclusion	✓
	12	The patient should share their perspective in one to two paragraphs on the treatment(s) they received	✗
**Informed Consent**	13	Did the patient give informed consent? Please provide if requested	✓ Yes No
